# Supplementary material for: Characterization of the Lipidome of Neurons in Mouse Brain Nuclei Using Imaging Mass Spectrometry
Source: Anal Chem. 2026 Jan 13;98(3):2579–88. doi: 10.1021/acs.analchem.5c08016 (PMC12856826; doi:10.1021/acs.analchem.5c08016)
Supplement: Supplementary file 1 [file ac5c08016_si_001.pdf]

Supporting Information

Characterization of the Lipidome of Neurons in Mouse Brain Nuclei using Imaging Mass Spectrometry

Cristina Huergo[1‡], Laura De las Heras-García[2,3,4‡], Jone Razquin[2,3,5], Yuri Rueda[6], Cristina Miguélez [*2,3‡] and José A Fernández[*1‡].

[1]Dep. of Physical Chemistry, Fac. of Science and Technology, University of the Basque Country (UPV/EHU), Barrio Sarriena S/N, 48940 Leioa, Spain; [2]Dep of Pharmacology, Faculty of Medicine and Nursing, University of the Basque Country (UPV/EHU), Barrio Sarriena S/N, 48940 Leioa, Spain; [3] Neurodegenerative Diseases, Biobizkaia Health Research Institute, Barakaldo, 48903, Spain; [4]Univ. Bordeaux, CNRS, IMN, UMR 5293, F-33000, Bordeaux, France.; [5]Dep. of Neuroscience, University of the Basque Country (UPV/EHU), Barrio Sarriena S/N, 48940, Leioa, Spain;[6] Department of Physiology, Faculty of Medicine and Nursing, University of the Basque Country (UPV/EHU), B. Sarriena, s/n, Leioa 48940, Spain

Index

INDEX

Figure S1. Experimental workflow S2
Figure S2. Relative abundance of PEe/PCe in LC, Me5 and SNc neurons S3
Figure S3. Relative abundance of PE/PC in LC, Me5 and SNc neurons S4
Figure S4. Relative abundance of lipid species in LC, Me5 and SNc neurons S5
Figure S5. Relative abundance of PE/PC and PEe/PCe in male/female LC neurons S6
Figure S6. Relative abundance of lipid species in male/female LC neurons S7
Figure S7. Relative abundance of PE/PC and PEe/PCe in male/female ME5 neurons S8
Figure S8. Relative abundance of lipid species in male/female ME5 neurons S9
Figure S9. Relative abundance of PE/PC and PEe/PCe in male/female ME5 neurons S10
Figure S10. Relative abundance of lipid species in male/female ME5 neurons S11
Table S1. Number of animals and sections used in this work S12

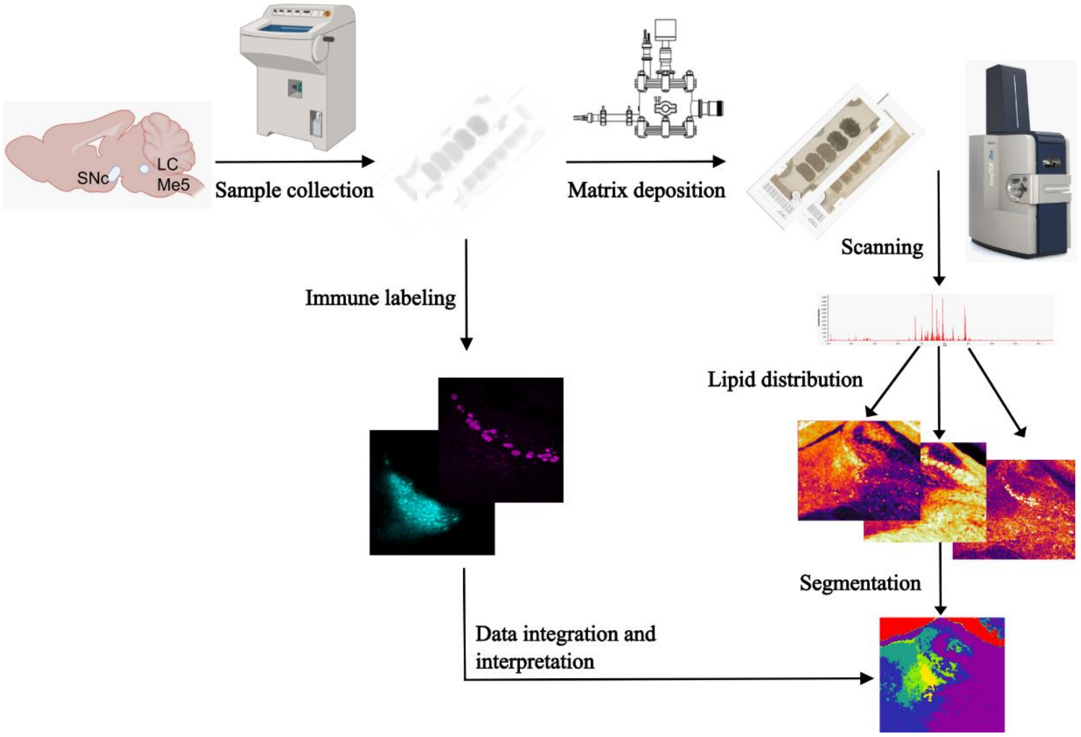

Figure S1. Experimental workflow. Sections of male and female mice brain were obtained with the aid of a cryo-microtome. Alternate sections were deposited in plain and ITO-coated microscope slides, so the imaging mass spectrometry (IMS) experiments were accompanied by immunofluorescence (if) images to certify the identity of the neurons. Segmentation was guided by the IF images.

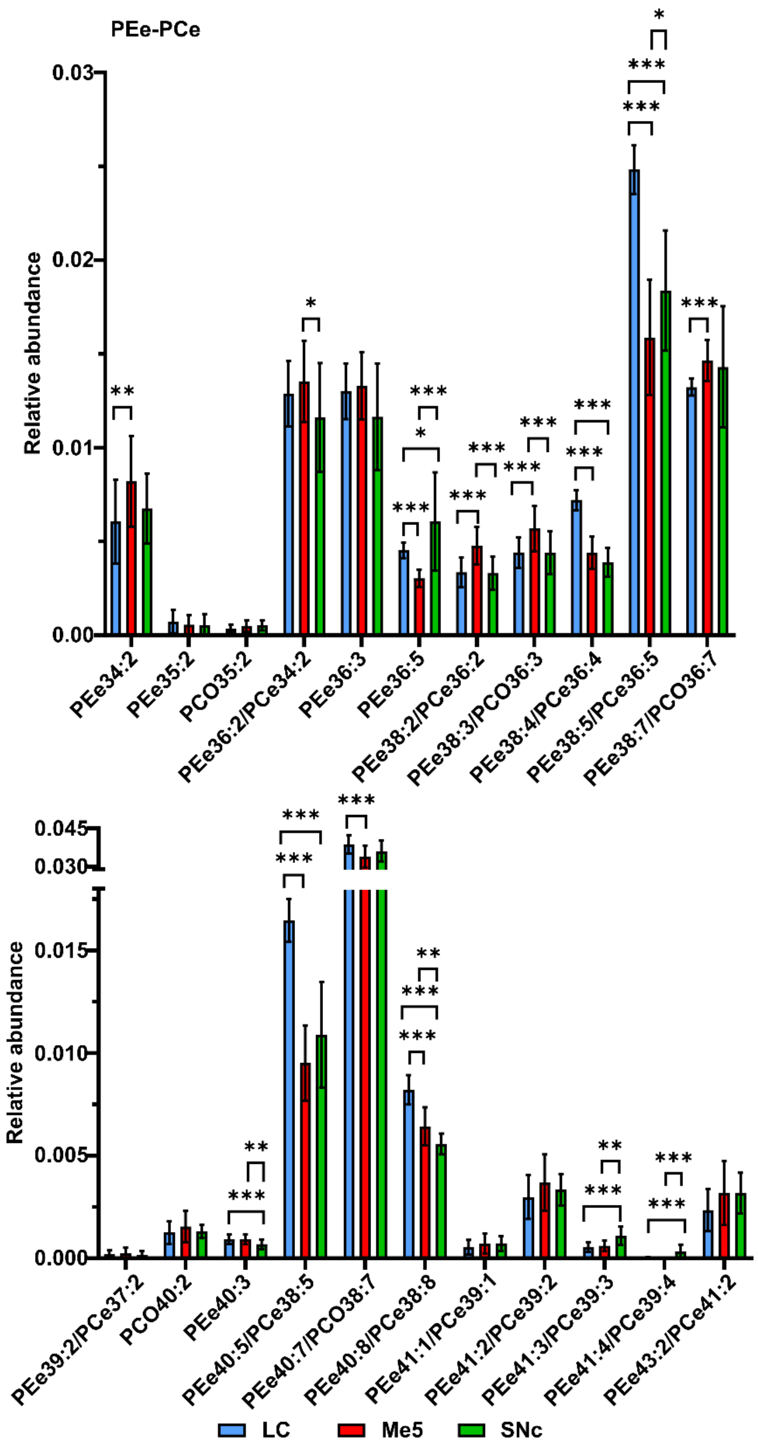

Figure S2. Comparison of the relative abundance of lipid species between the ROIs containing the neurons of the LC, Me5 and SNc. PE and PC are shown together due to the overlap of several species in the same m/z. The comparison was divided into two panels for clarity. * = p<0.05, ** = p<0.01, *** = p<0.001

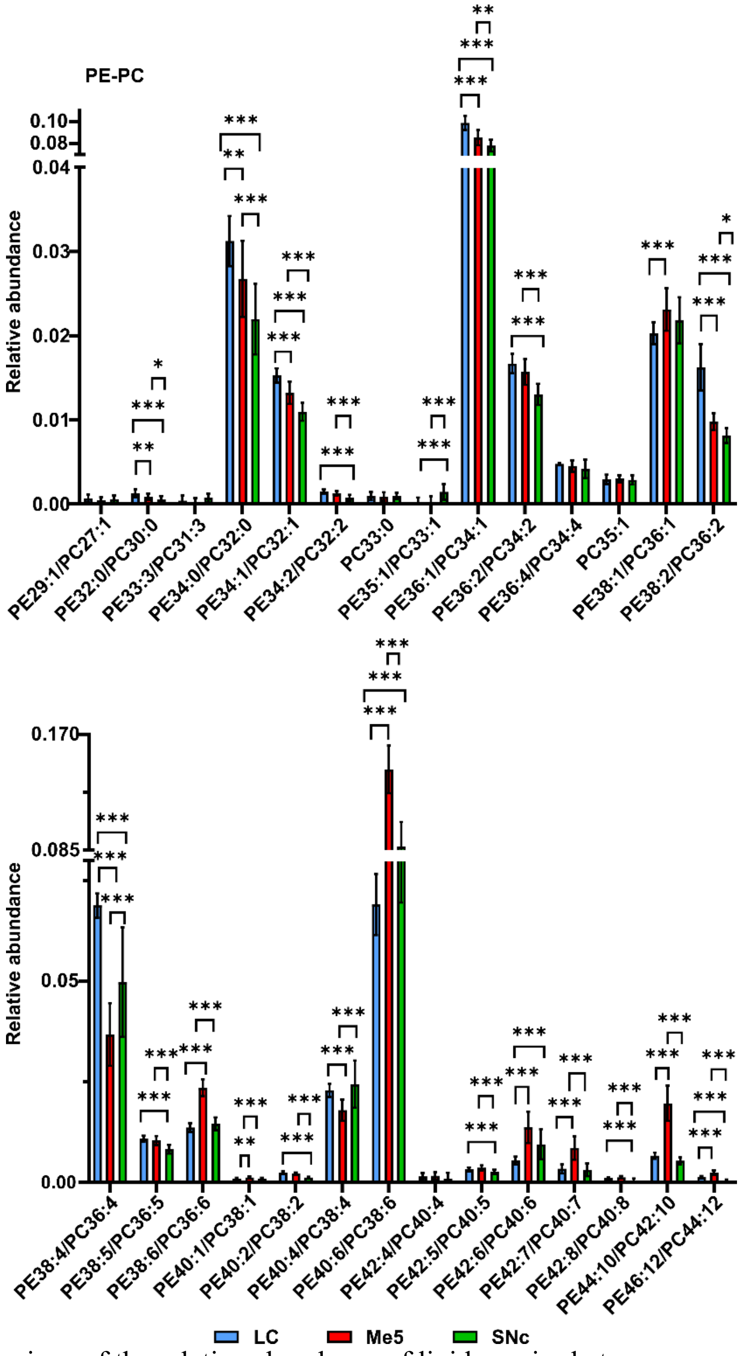

Figure S3. Comparison of the relative abundance of lipid species between neurons of the LC, Me5 and SNc. PEe and PCe are shown together due to the overlap of several species in the same m/z. PEe/PCe was divided into two panels for clarity. * = $p<0.05$, ** = $p<0.01$, *** = $p<0.001$.

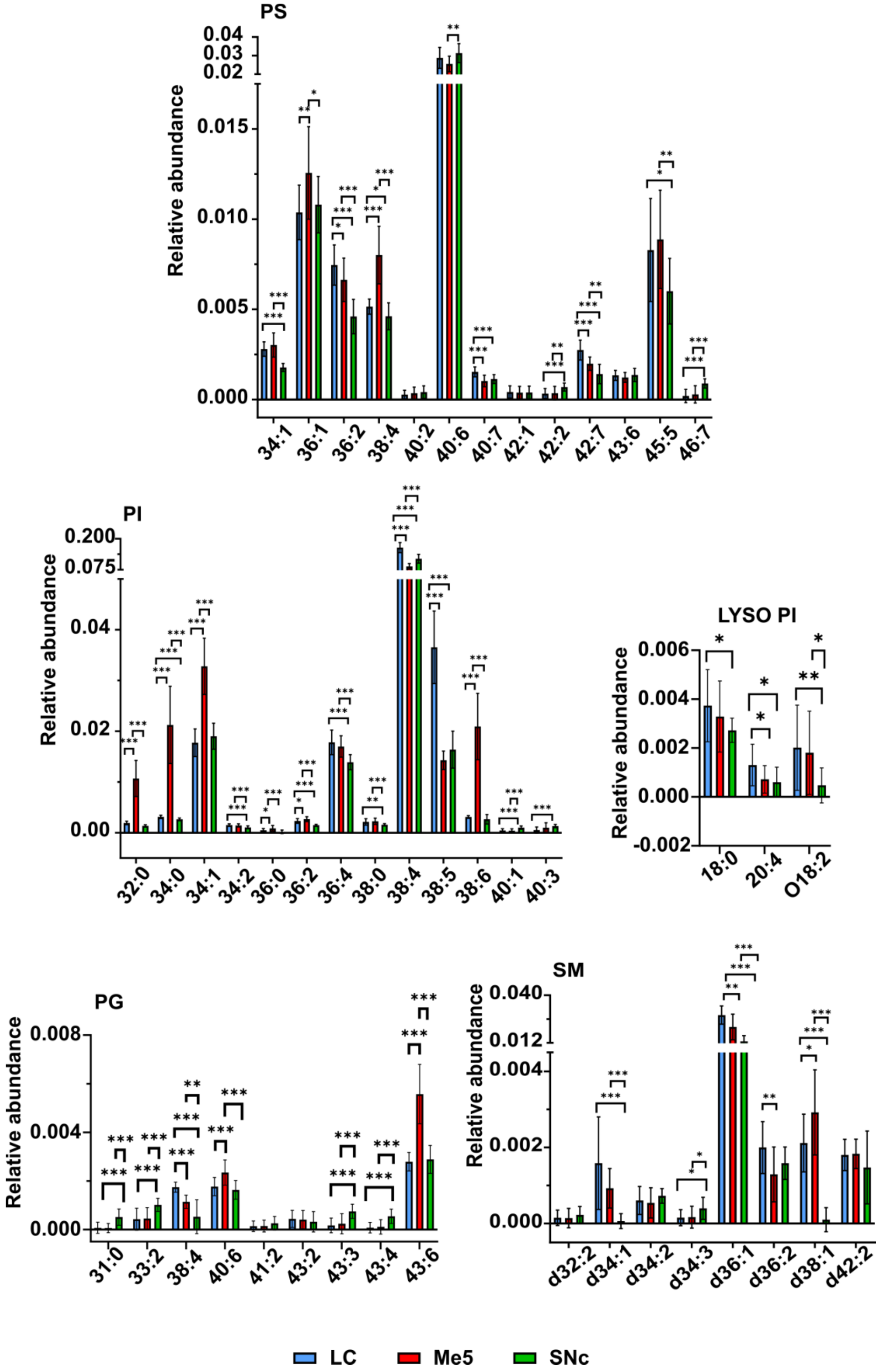

Figure S4. Comparison of the relative abundance of lipid species between neurons of the LC, Me5 and SNc. * = $p<0.05$, ** = $p<0.01$, *** = $p<0.001$.

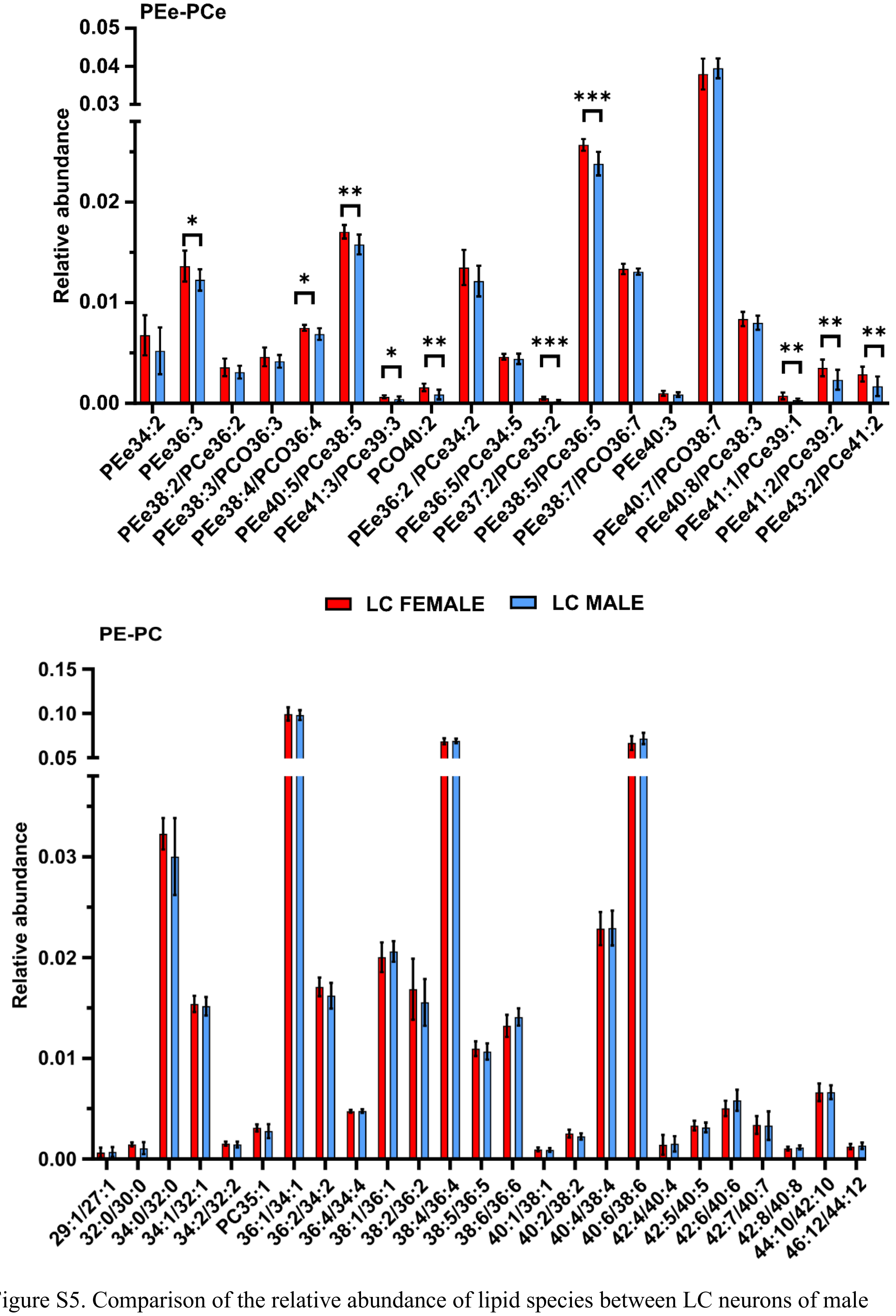

Figure S5. Comparison of the relative abundance of lipid species between LC neurons of male and female mice. PE and PC and PEe and PCe are shown together due to the overlap of several species in the same m/z. * = $p<0.05$, ** = $p<0.01$, *** = $p<0.001$.

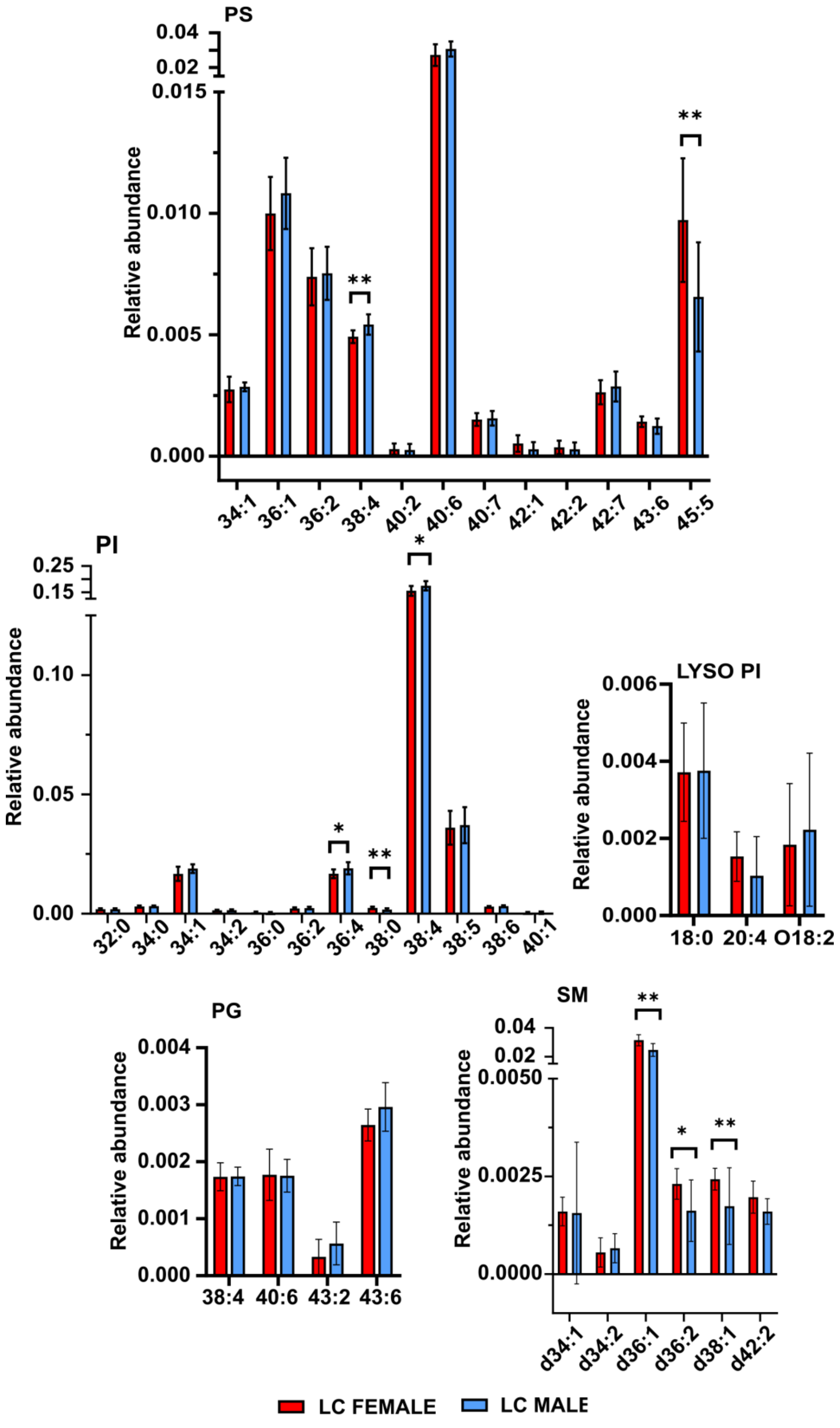

Figure S6. Comparison of the relative abundance of lipid species between LC neurons of male and female mice. For the rest of the classes see Figure S5. * = p<0.05, ** = p<0.01, *** = p<0.001.

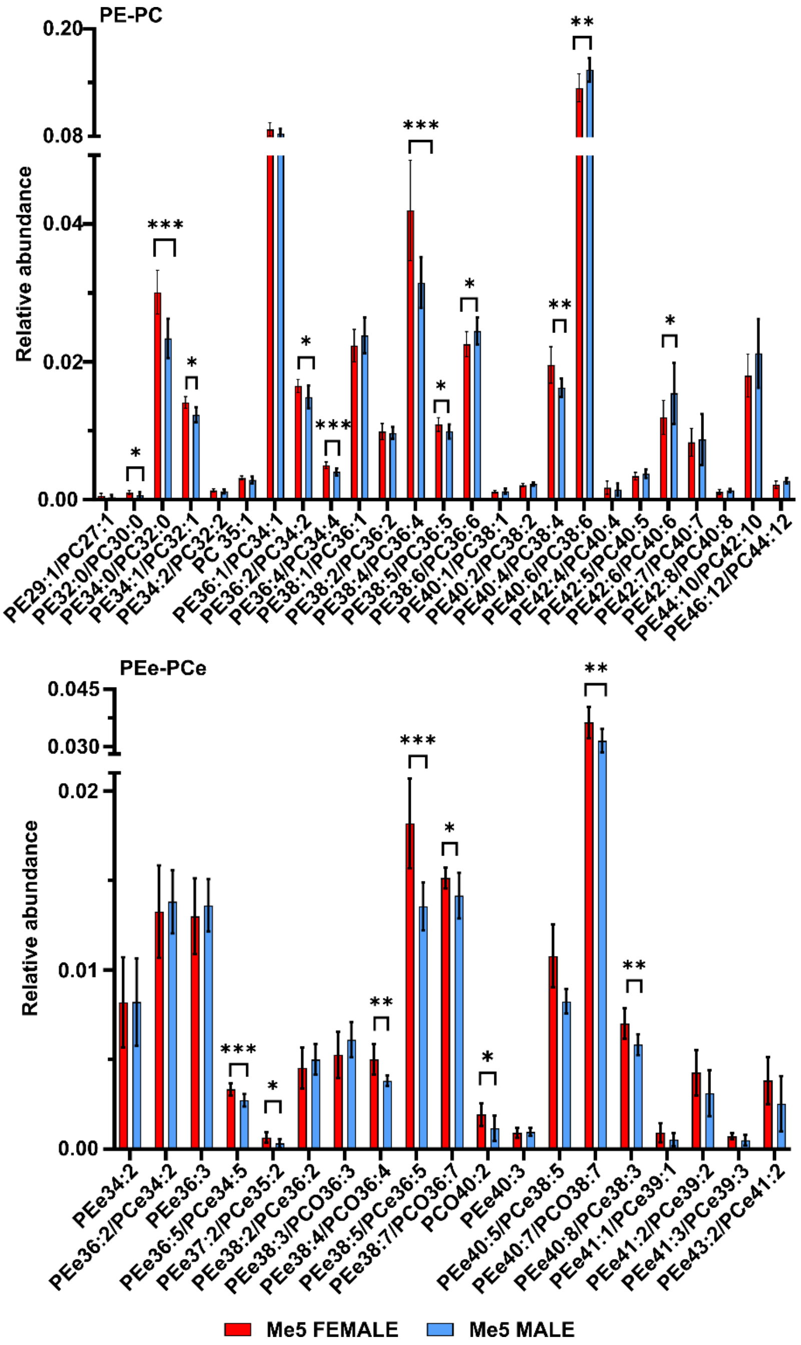

Figure S7. Comparison of the relative abundance of lipid species between Me5 neurons of male and female mice. PE and PC are shown together due to the overlap of several species in the same m/z. PEe and PCe were also grouped for the same reason. * = $p<0.05$, ** = $p<0.01$, *** = $p<0.001$.

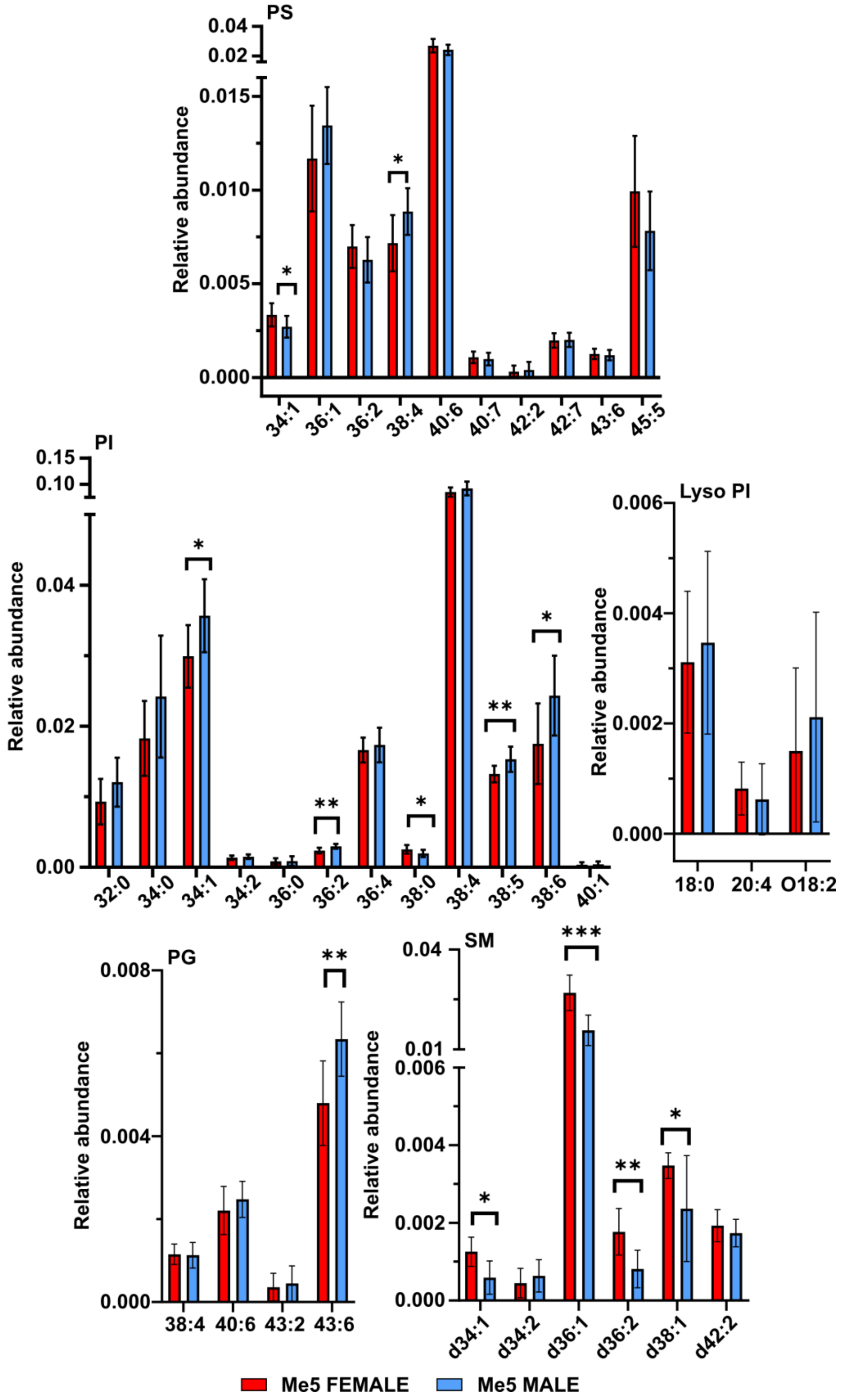

Figure S8. Comparison of the relative abundance of lipid species between Me5 neurons of male and female mice. For the rest of the classes see Figure S7. * = $p<0.05$, ** = $p<0.01$, *** = $p<0.001$.

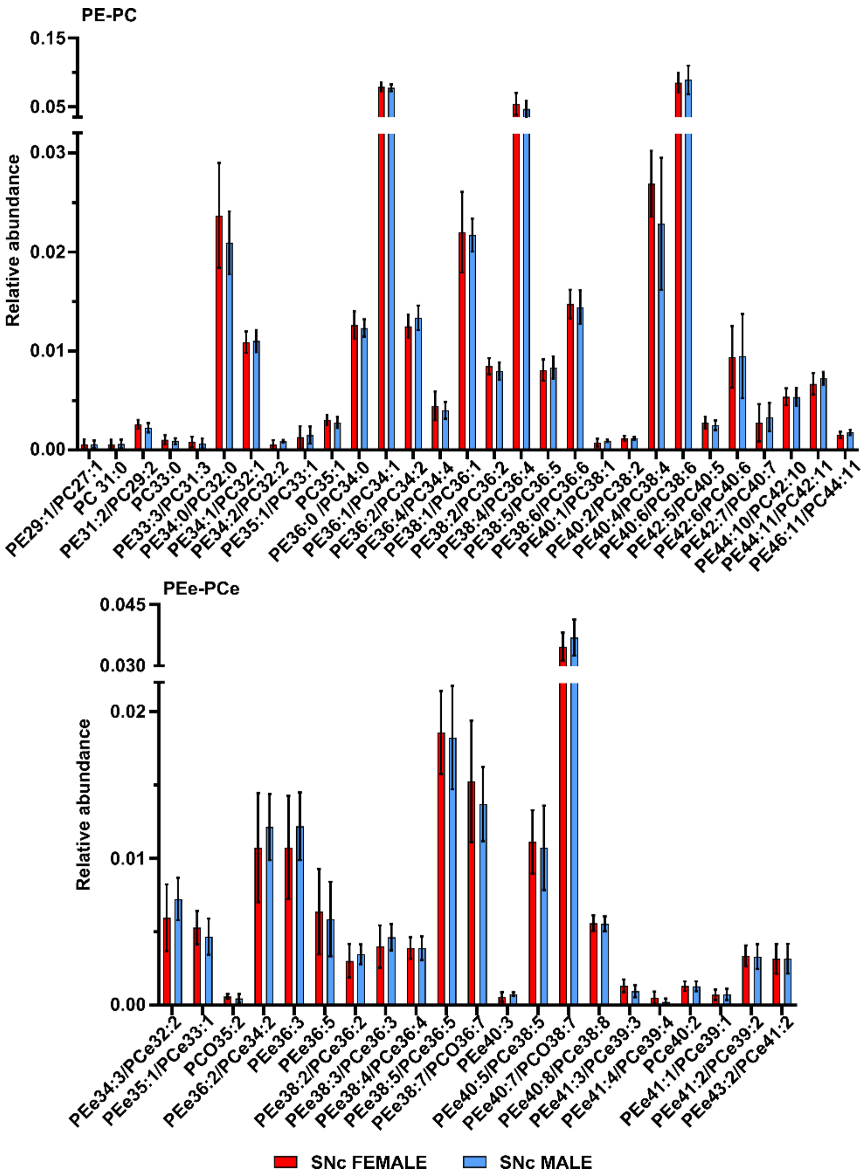

Figure S9. Comparison of the relative abundance of lipid species between SNc neurons of male and female mice. PE and PC are shown together due to the overlap of several species in the same m/z. PEe and PCe were also grouped for the same reason. For the rest of the classes see Figure S10. * = p<0.05, ** = p<0.01, *** = p<0.001.

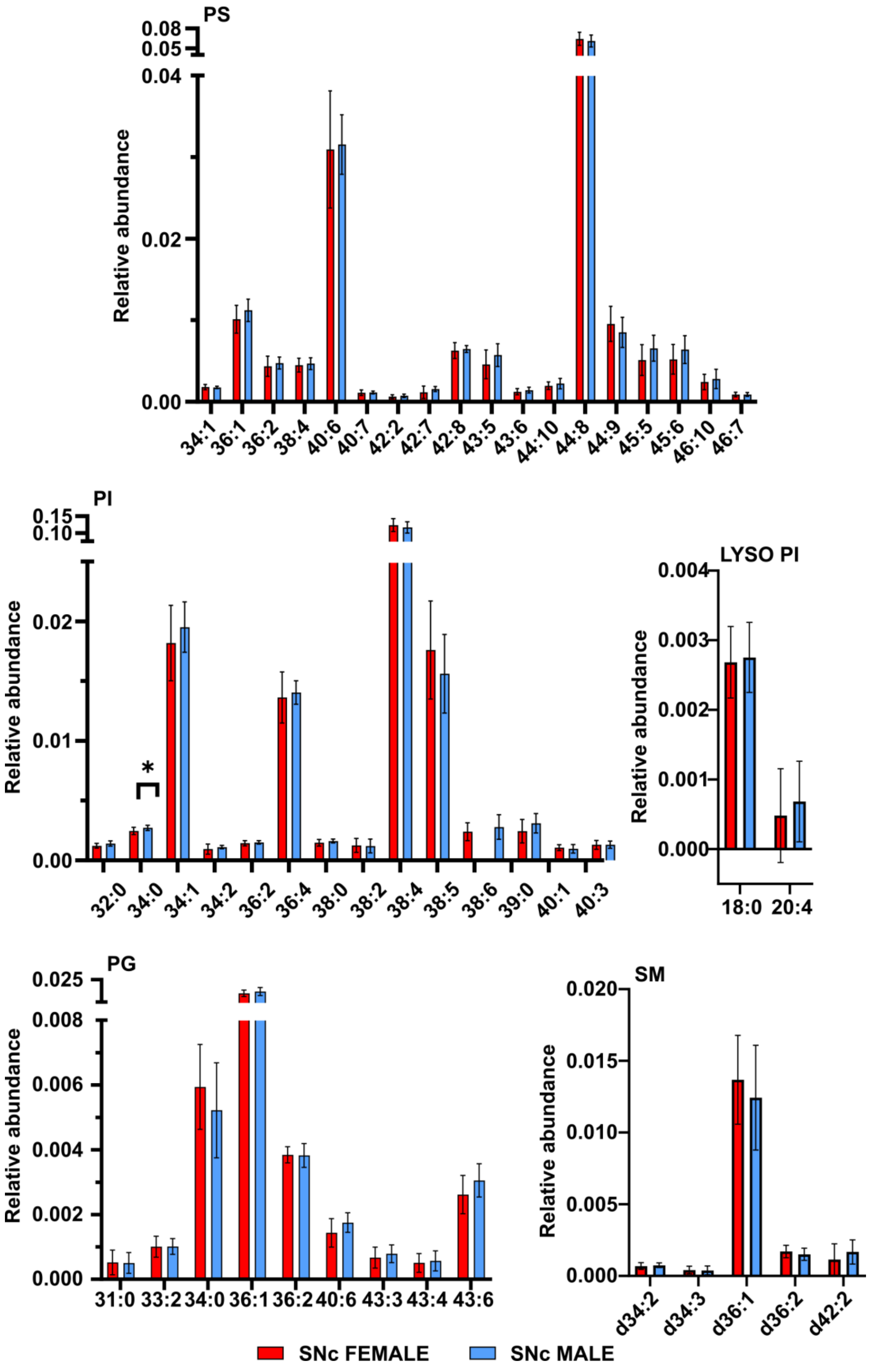

Figure S10. Comparison of the relative abundance of lipid species between MSN neurons of male and female mice. For the rest of the classes see Figure S9. * = p<0.05, ** = p<0.01, *** = p<0.001.

Table S1. Number of animals and sections used in this work. Some of the sections scanned contained no LC, ME5 or SNc and therefore, the final number of ROIs used for the analysis is different from the number of regions scanned.

	nº animals	sex	nº	nº sections	measured regions	regions used/neuron type
LC	10	female	5	26	52	50
		male	5	26	52	40
Me5	10	female	5	26	52	26
		male	5	26	52	20
SN	12	female	6	34	68	10
		male	6	32	64	11
